# Passive Electrical Damping of a Quartz Tuning Fork as a Path to Fast Resonance Tracking in QEPAS

**DOI:** 10.3390/s21155056

**Published:** 2021-07-26

**Authors:** Roman Rousseau, Diba Ayache, Wioletta Trzpil, Michael Bahriz, Aurore Vicet

**Affiliations:** IES, University Montpellier, CNRS, 34090 Montpellier, France; Roman.Rousseau@ies.univ-montp2.fr (R.R.); diba.ayache@ies.univ-montp2.fr (D.A.); Wioletta.Trzpil@ies.univ-montp2.fr (W.T.); michael.bahriz@umontpellier.fr (M.B.)

**Keywords:** quartz tuning fork, gas sensor, photoacoustic, beat frequency

## Abstract

In Quartz-Enhanced PhotoAcoustic Spectroscopy (QEPAS) gas sensors, the acoustic wave is detected by the piezoelectric Quartz Tuning Fork (QTF). Due to its high-quality factor, the QTF can detect very low-pressure variations, but its resonance can also be affected by the environmental variations (temperature, humidity, …), which causes an unwanted signal drift. Recently, we presented the RT-QEPAS technique that consistently corrects the signal drift by continuously measuring the QTF resonance. In this article, we present an improvement of RT-QEPAS to fasten the QTF characterization time by adding a passive electronic circuit, which causes the damping of the QTF resonance. The damping circuit is optimized analytically and through SPICE simulation. The results are supported by experimental observations, showing a 70 times improvement of the relaxation times compared to the lone QTF, which opens the way to a fast and drift-free QEPAS sensor.

## 1. Introduction

Originally presented in 2002 by Kosterev et al., QEPAS (Quartz-Enhanced PhotoAcoustic Spectroscopy) is now a well-established technique in gas sensing [1]. As in photoacoustics, a modulated laser is used to excite a gas species in order to create a sound wave thanks to the photoacoustic effect. Then, the acoustic energy is converted to an electrical signal by the piezoelectric quartz tuning fork (QTF).

QEPAS gas sensors have been employed in diverse applications, for instance, in air pollution monitoring [2], engine exhaust measurements [3], breath analysis [4], and biogas production [5]. Depending on the application, the environmental conditions (temperature, pressure, humidity) and the composition of the sample can greatly vary. Those variations might affect the QEPAS sensor at different stages. Certainly, the most significant source of error, or signal drift, is the variation of the QTF resonance, i.e., both the resonant frequency *f*_0_ and the quality factor *Q*. Indeed, the QTF has a very sharp resonance and is, therefore, highly sensitive to the surrounding environment.

In order to prevent the signal drift, mostly two methods have been suggested in the literature. The first one is based on the measurement of the QTF instantaneous frequency change by using an oscillator circuit. The sensor has a good stability and a fast response time [6]. This method is promising but still under development. The second method is called RT-QEPAS [7] and consists of the regular characterization of the QTF to apply a correction onto the laser modulation frequency. RT-QEPAS is a simple method that does not require major modifications of the conventional QEPAS setup. RT-QEPAS integrates into the QEPAS sensing process, a sequence of resonant frequency, and quality factor measurement of the QTF, by measuring the electrical relaxation of the QTF after a short excitation. For instance, we were able to demonstrate a significant reduction of the drift for the detection of wet CH_4_. However, the main limitation of RT-QEPAS is the time spent for characterizing the QTF. Indeed, after the electrical excitation, the QTF undergoes an exponential decay. It takes about 1 s to recover an oscillation amplitude that does not affect the QEPAS measurement. A priori, the relaxation time is an intrinsic behavior of the QTF that cannot be modified without altering the sensitivity of the gas sensor. Nonetheless, it is possible to employ an external harmonic oscillator to reduce the damping time.

Probably the most famous example of passive vibration damping is the Taipei 101 skyscraper. In the tower, which can be represented as a spring-mass-damper system, a giant steel ball, weighing 660 tons, is implemented as a tuned mass damper in order to reduce the vibrations in the building [8,9]. In a very similar manner, we will employ an additional resonant electrical circuit, we called the damping circuit, to reduce the oscillations of the QTF.

In Section 2, the electronic design of the damping circuit is presented, and the decay time of the system is minimized through an analytical study. Then, in Section 3, the transient response of the damped QTF effect is studied while varying the damping resistance and the damping capacitance. The transient response is simulated using a SPICE software, and the results are experimentally validated. In Section 4, another circuit based on a MOSFET is added to provide an active control of the damping. Finally, we present the proof of concept of the RT-QEPAS technique implemented with the damping circuit in Section 5.

## 2. Theoretical Optimization

The QTF is a mechanical harmonic oscillator. The moving parts can be represented as a simple mass-spring system; the mechanical losses are accounted by the equivalent damper. In order to increase the losses of the system, another mechanical damper could be added in series and, therefore, obtain the desired damping effect.

**Figure 1:**
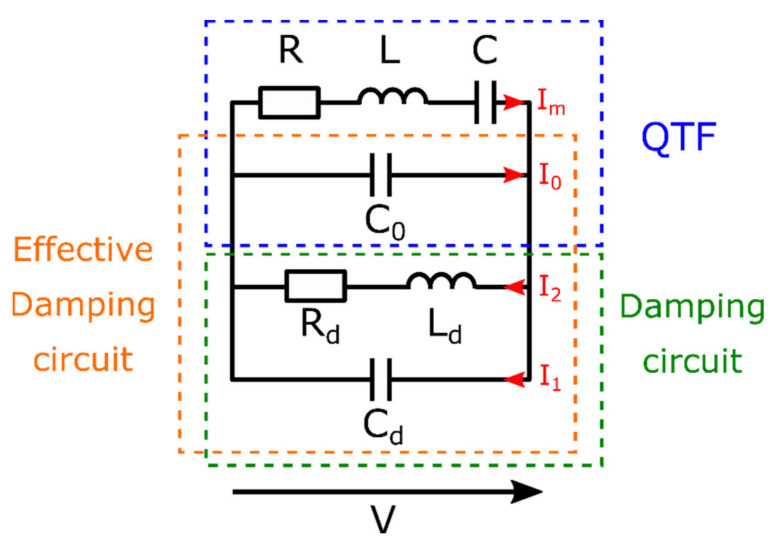
Schematic of the Quartz Tuning Fork (QTF) and the damping circuit. (*R* = 100 kΩ, *L* = 6 kH, *C* = 4 fF, *C*_0_ = 2 pF).

Due to its piezoelectric feature, the QTF motional behavior is equivalently represented by a series RLC circuit (mass ⇔ inductance *L*, spring ⇔ capacitance *C*, damper ⇔ resistance *R*). However, in the electrical equivalent circuit there is an additional parallel term, *C*_0_, which is due to the capacitance formed by the electrodes and the quartz material. The equivalent circuit is called the Butterworth Von-Dyke model (blue dotted square in Figure 1) [10]. In this circuit, the parallel capacitance prevents the possibility to physically add an electrical damper in the RLC branch. If the QTF is loaded with a resistance *R_d_*, the energy stored in the motional RLC will flow into the capacitance *C*_0_, instead of being absorbed by *R_d_*. Therefore, we concluded that a simple resistor cannot interact with the QTF. Instead, we designed a damping circuit based on a complex impedance: a parallel RLC circuit (green dotted square in Figure 1). The newly formed RLC circuit is made of one branch comprising a damping resistor (*R_d_*), a damping inductance (*L_d_*), in parallel with another branch with a damping capacitance (*C_d_*). Since the damping capacitance (*C_d_*) is in parallel with the QTF capacitance *C*_0_, the effective damping capacitance is the sum of the two contributions (*C_d_* + *C*_0_).

In order to obtain the damping effect, the parameters must be finely adjusted. The analytical study we present here focuses on the optimization of the damping parameters. It is based on a similar work [11] in which a piezo patch is mechanically bonded to a cantilever to reduce its movement through passive electrical damping. Many other relevant resources can be found in the field for solving such problems [12,13,14].

The electrical circuit presented in Figure 1, leads to a system of coupled differential equations:

(1) $$L{\ddot{q}}_{m}+R{\dot{q}}_{m}+\frac{1}{C}q_{m}+V=0$$

(2) $$L_{d}{\ddot{q}}_{2}+R_{d}{\dot{q}}_{2}-V=0$$

with ${\dot{q}}_{m}=I_{m}$ and ${\dot{q}}_{2}=I_{2}$.

The details of the calculation can be found in [15]. Notably, the system is re-written and let appear important parameters such as the mechanical resonant frequency of the QTF $\omega_{m}$ and the resonant frequency of the (effective) damping circuit $\omega_{d}$:

$$\omega_{m}=2\pi f_{0}=\frac{1}{\sqrt{LC}},\;\;\;\omega_{d}=\frac{1}{\sqrt{L_{d}C_{0}\left(1+\alpha \right)}},\;\;\;\alpha =\frac{C_{d}}{C_{0}},$$

with α the capacitance ratio (which is convenient for expressing the optimum parameters).

Then, the system is optimized in order to minimize to the decay time. The first and most important result of the optimization is the frequency match of the damping circuit with the QTF. The optimum damping is obtained at resonance: $\omega_{m}=\omega_{d}$. This condition is met for a given value of the inductance $L_{d,opt}$:

(3) $$L_{d,opt}=L\frac{C}{C_{0}\left(1+\alpha \right)}$$

The second result of the optimization is the value of the electrical dissipation of the damping circuit, which ultimately leads to the optimal value of the damping resistance $R_{d,opt}$:

(4) $$R_{d,opt}=\frac{2\sqrt{LC}}{C_{0}\left(1+\alpha \right)}$$

When the optimal conditions ($L_{d,opt}$ and $R_{d,opt}$) are met, the 1-decade decay time reaches its minimal value and can be expressed as:

(5) $$\tau_{d,\;opt}=\;\frac{2\ln\left(10\right)}{\omega_{m}}\;\sqrt{\frac{C_{0}\left(1+\alpha \right)}{C}}=\;2\ln\left(10\right)\sqrt{LC_{0}\left(1+\alpha \right)}$$

**Figure 2:**
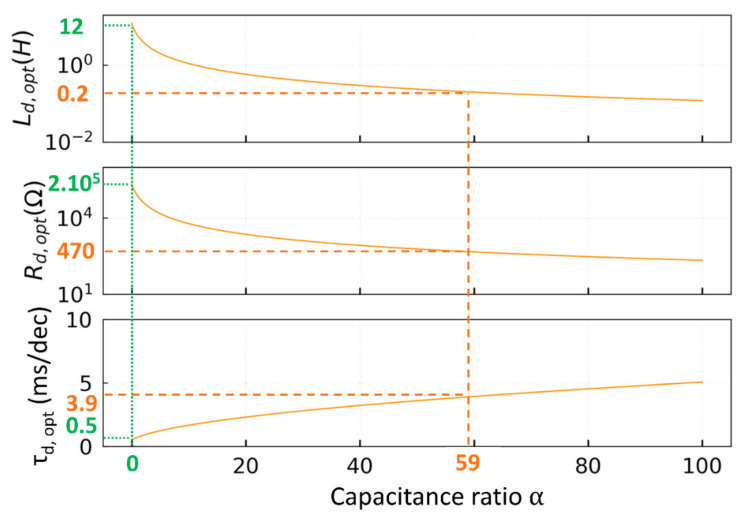
Evolution of the optimum damping inductance, resistance and 1-decade decay time as a function of *α*. The minimum decay time of 0.5 ms/dec is obtained for a capacitance ratio of 0 (green dotted line). In our case, for a damping inductance of 0.2 H, the capacitance ratio is 59, which leads to an optimal damping resistance of 470 and a decay time of 3.9 ms/dec as shown by the orange dotted line.

Using Equations (3)–(5), the optimum parameters can be plotted in Figure 2 as a function of *α*. *R_d,opt_* and *L_d,opt_* are decreasing with *α* while the decay time *τ_d,opt_* increases. The decrease of *L_d,opt_* with increasing *α* (equivalently *C_d_*), is necessary to ensure the resonant condition of the damping circuit with the QTF ($\omega_{m}=\omega_{d}$). The variation of *R_d,opt_* can be understood in terms of losses. A damping circuit with low losses will not quickly absorb the charges from the QTF, while a damping circuit with high losses will not interact with the QTF efficiently as the charges will bypass the RL branch through the parallel capacitance. Therefore, *R_d_* has an optimum value, which appears to be inversely proportional to the damping capacitance. Oppositely, the decay time is proportional to the square root of alpha.

The shortest damping time *τ_d_* is obtained when the capacitance ratio is zero, i.e there is no additional damping capacitance (*C_d_* = 0). However, it requires a damping inductance of 12 H, which simply means a large coil physically speaking. In order to lower *L_d_*, we have to increase the capacitance ratio, which implies an increase of the damping time. However, *τ_d_*. is proportional to the square root of *α*, so the time loss is progressive. We chose an inductance of 0.2 H, as a good compromise of component size and damping time which leads to a capacitance ratio of 59. The optimal resistance $R_{d,opt}\;$ is around 500 Ω, which happens to correspond to the intrinsic resistance of the coils in the range of 0.2 H. For *α* = 59, the decay time is about 4 ms/dec, only about ten times longer than the optimum value when *α* = 0, but already *about 70 times faster than the QTF spontaneous decay* ($\tau_{d}=\tau \ln\left(10\right)=2\ln\left(10\right)\frac{L}{R}=280$ ms/dec).

Thanks to this analytical study, we were able to optimize the damping circuit and to estimate the damping time. Nonetheless, we lack any information concerning the decay time for non-optimal value of *L_d_* and *R_d_*. Indeed, since the damping circuit is resonantly pulling the energy out of the QTF, the variation of the damping capacitance or inductance would lead to a shift of the damping resonance and a longer decay time. In this regard, the SPICE simulation provides complementary information to the analytical study.

## 3. Effect of the Damping Resistance and the Damping Capacitance

In this section, we study the transient response of the damped QTF effect while varying the damping resistance and the damping capacitance. The transient response was simulated using a SPICE software (OrCAD PSPICE) [16] and the results were validated experimentally.

Before all, we should underline the difficulty of simulating the QTF transient response due to its high-quality factor (the time constant to oscillation period ratio (*τ/T* = *Q*/π ≈ 4000) is very high). On the one hand, the discrete timestep must be small enough compared to the rate of change of the circuit, for instance much lower than the QTF oscillation period. On the other hand, the simulation time must be larger than the QTF time constant. A small timestep and a large duration means a large dataset. Practically, for a timestep of 200 ns and a simulation time of 1 s, 5 million data points are required. For the study of the damped QTF, the decay is faster than the undamped QTF and we were able to obtain significant results with a simulation time of only 50 ms, i.e., only about 250 k data points.

**Figure 3:**
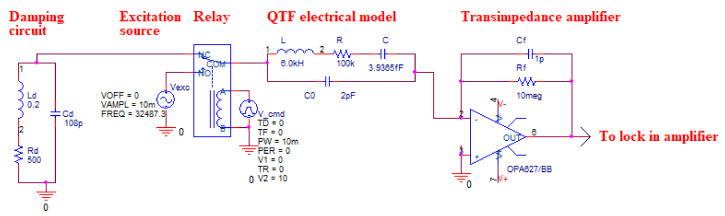
Schematic of the electronics circuit on OrCAD PSPICE including the damping circuit, the excitation source, the switching relay, the QTF and the transimpedance amplifier [7].

The schematic of the simulated circuit is shown in Figure 3. It includes the damping circuit, a sinewave voltage source, an electro-mechanical relay, the QTF equivalent model and a transimpedance amplifier (TA). The effect of the damping circuit onto the QTF is well described by displaying the QTF energy (Li^2^(t) + Cu^2^(t)) or the output voltage of the amplifier. The latter is more relevant because it is the one practically measurable. Experimentally, the measurements are performed using a lock-in amplifier (LIA) (Zurich Instruments MFLI, Zurich, Switzerland) located after the transimpedance amplifier. The time constant of the LIA was set around 1 ms in order to measure the beat signal [7].

**Figure 4:**
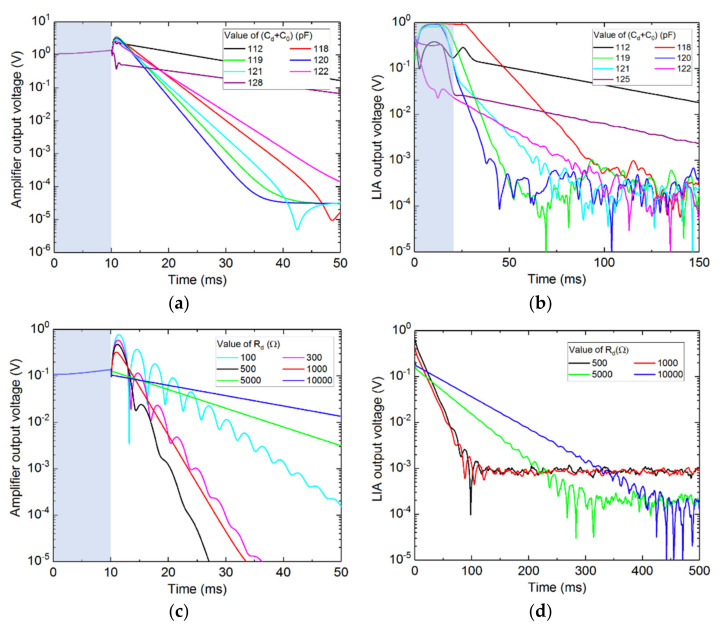
Simulated (left) and experimental (right). QTF response with the damping circuit while varying the damping capacitance (**a**,**b**) (with *R_d_* = 500 Ω) and the effective damping resistance (**c**,**d**) (with *C_d_* + *C*_0_ = 120 pF). The electrical excitation takes place during about 10 ms as represented by the blue area. Then, the electrical excitation is stopped and the QTF is connected to the damping circuit. For the experiments, the QTF employed was a NC38LF (Fox Electronics, Fort Myers, FL, USA), with a resonant frequency of 32,757 Hz and a quality factor of about 11,000.

In order to observe the effect of the damping circuit onto the QTF, a transient simulation was carried out: first, the QTF is excited by the excitation source during 10 ms (blue area in Figure 4). Then, the relay switches and the damping circuit starts interacting with the QTF. The results for both the simulation and the experiment are compared in Figure 4. As expected, the fastest decay is obtained when the QTF and the damping circuit are resonantly matched, corresponding to *C_d_* + *C*_0_ = 120 pF theoretically (Figure 4a) as well as experimentally (Figure 4b). In this experiment, *C*_0_ was measured to be 12 pF, which differs from the theoretical value of 2 pF, due to additional capacitance of the cables and the breadboard. Small variations of less than 1 pF greatly affect the damping effectiveness, confirming the importance of eliminating any parasitic capacitance. Illustrating the troubles we encountered, we first used a variable capacitor in order to tune *C_d_*. Upon touching the variable capacitor adjustment screw with a screwdriver, a parallel capacitance of a few pF was added to the system, shifting the resonance, and thus hindering the damping effect.

In terms of damping resistance, at low values, an interference pattern can be observed (Figure 4c), which can be understood as a periodic energy transfer between the QTF and the damping circuit due to a small resonance mismatch. The optimum decay time is simulated for a resistance around 500–1000 kΩ. Higher *R_d_* values lead to less damping, as observed experimentally (in Figure 4d).

**Figure 5:**
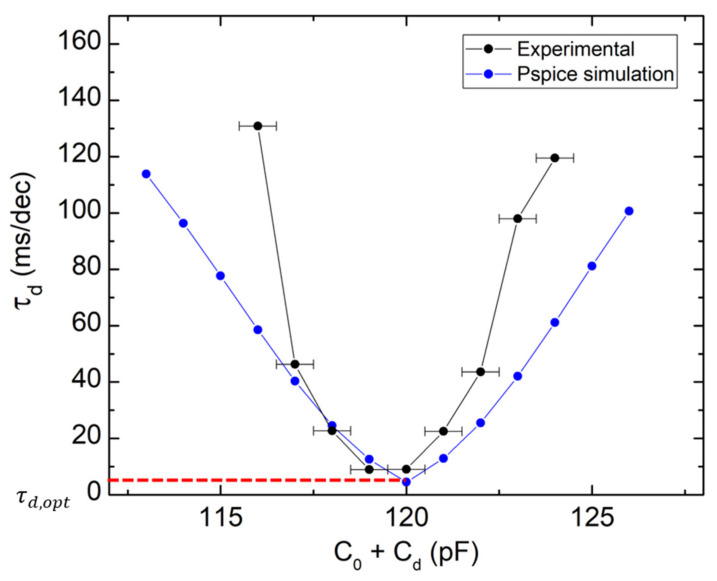
Evolution of the decay time as a function of the effective damping capacitance, comparing the experiment (black dots) with the PSPICE simulation (blue dots). The optimum decay time is of 9.0 and 4.5 ms/dec, for the experiment and the simulation, respectively.

As we focus on the damping effect, it is more convenient to represent the same results in terms of decay time (Figure 5). The 1-decade decay time *τ_d_* is the inverse of the slope on a logarithmic scale, and represents the time spent for a 1-decade signal drop. It follows a parabolic behavior as a function of the damping capacitance with an optimum value *τ_d,opt_* corresponding to the minimum value of the parabola. The position of the parabola vertex is similar for the experiment and the simulation. However, the experiment is more sensitive (i.e., with a higher curvature) to the effective damping capacitance than the simulation. It is certainly due to the error in the measurement of *C*_0_. The measured value is probably larger than the true value due to the parasitic capacitances of the characterization circuit.

Through the SPICE simulations and the experiments, we have been able to confirm the theoretical optimization of the damping inductance and resistance. Furthermore, we have seen that the variations of the damping capacitance greatly affect the damping and must be adjusted with an accuracy below the picofarad.

In order to fully exploit the damping effect in a QEPAS experiment, we must be able to activate the damping when required. Therefore, we added a MOSFET device for the active control of the damping circuit.

## 4. Control of the Damping Circuit with a MOSFET

Initially, we employed an electromechanical relay as a switching device to alternate between the QTF excitation and relaxation (Figure 3). However, the relay was generating an electrical surge during switching which provoked a re-excitation of the QTF. In this section, we present an attempt to lower the switching parasitic excitation by replacing the mechanical action of the relay with a purely electrical switching based on a MOSFET device.

**Figure 6:**
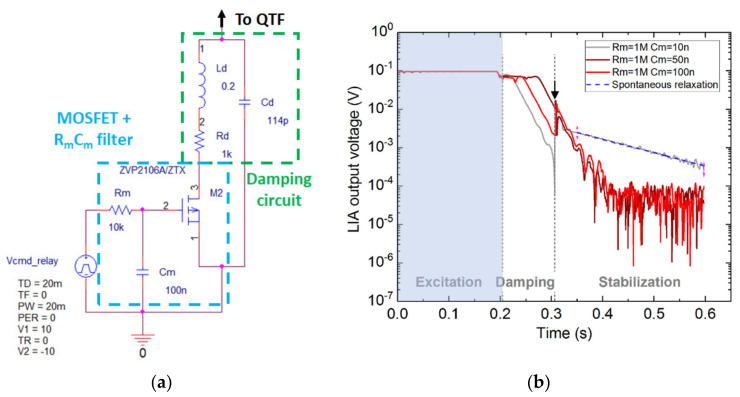
(**a**) PSPICE schematic of the damping circuit and the MOSFET inserted in the RL branch. The MOSFET gate is driven by a voltage source connected to a low pass RC filter. (**b**) Observing the QTF response to damping for different values of the filter capacitance *C_m_*. The damping is enabled just after the end of the excitation by applying a negative voltage to the MOSFET gate. The arrows indicate the moment the MOSFET was turned off, that is 100 ms after the onset of the damping.

After the QTF is discharged, the damping circuit needs to be disconnected from the QTF without re-exciting it, which was not feasible using the relay. The MOSFET can only be used as a two-port switch. It cannot completely replace the relay but can be employed when the relay failed to disconnect ‘silently’ the damping circuit. To this end, we placed the transistor inside the RL branch of the damping circuit (Figure 6), thus obtaining a voltage-controlled damping resistor.

The damping circuit is driven by the voltage at the MOSFET gate (V_GS_). When the MOSFET is polarized, charges accumulate inside the gate. The charges might leak into the source upon changing the polarization. In order to smooth this parasitic effect and avoid QTF re-excitation, a low pass *R_m_C_m_* filter was added before the MOSFET gate. The resistance *R_m_* is chosen to be 1 MΩ to have a low gate current. To adjust the triggering delay of the MOSFET, different capacitance values *C_m_* were tested and the response of the QTF with the damping circuit was observed (Figure 6b).

The QTF is first excited, then discharged by the damping circuit and finally the damping circuit is disabled and the QTF is let free for stabilization. When *C_m_* is low (grey), the RC filter constant is small, so is the smoothing effect of the low pass filter, causing the QTF to be re-excited by the fast pulse, as indicated by the slowly decaying slope driven by the QTF spontaneous decay (blue dotted line). When *C_m_* is ideally chosen (brown and red), the pulse does not provoke the QTF re-excitation and allows to maintain the desired damping effect. It can be seen that the signal reached the noise floor (10^−4^–10^−5^ V), about 200 ms after the onset of the damping.

### Partial Damping Using the Switching Time

In order to prove the effectiveness of the MOSFET-based active control and to further exploit the damping effect, we realized a partial damping, by controlling the damping time.

**Figure 7:**
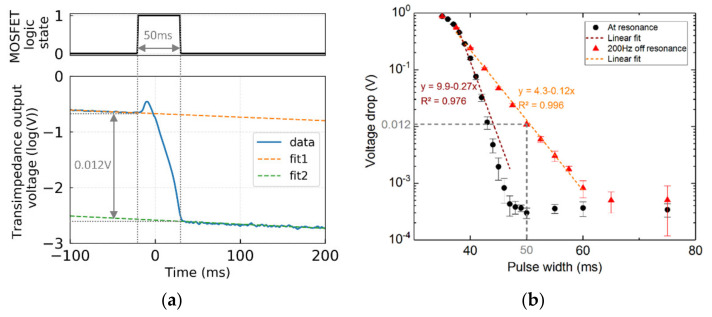
(**a**) Example of the fitting procedure for a pulse width of 50 ms. The log-plotted data are linearly fitted before damping (yellow dashed line) and just after the damping (green dashed line). The voltage drop is obtained by subtracting the intercept of the two linear curves. (**b**) Voltage drop, measured at the output of the transimpedance amplifier (TA), as a function of the pulse width sent to the MOSFET gate. The experiment was realized at resonance (black dots) and 200 Hz off resonance (red triangles). The errors bars represent the standard deviation of the 10 measurements from Figure 7a.

Using a function generator, a pulse of a given width was sent to the MOSFET gate to activate the damping circuit for a precise duration (for example, 50 ms in Figure 7a, top graph). In the meantime, the voltage drop at the output of the transimpedance amplifier was recorded (Figure 7a, bottom graph). The data are plotted on a log scale and linearly fitted (corresponding to the QTF exponential decay) before and after the damping, yielding the voltage drop between the two curves intercepts (here, 0.012 V). The measurement from Figure 7a was repeated 10 times for each pulse duration, from which were extracted the mean value and the standard. The results are presented in Figure 7b.

When the damping circuit is chosen to be resonant with the QTF (round black dots), the slope is the highest (in absolute value), confirming the QTF fast discharge. The slope is around 3 ms/decade, as obtained in optimal conditions (Figure 2). In this first case, i.e., at resonance, the error bars are significant. Although this issue could possibly be circumvented with a deeper study of the electronics of the damping system, we found another solution to improve the accuracy of the partial damping by tuning the damping circuit about 200 Hz off resonance (square red dots) (corresponding to a shift of *C_d_* by 1.5 pF off the optimal value). In this second case, the damping time is increased (with a slope of 8 ms/dec), but the errors are greatly reduced. A complete study would be useful to have more quantified values, but it won’t be detailed in this paper. Moreover, the curve exhibits a very good linearity (*R* = 0.996 instead of 0.976 for the resonant case), thus obtaining a direct proportionality between the damping time and the voltage drop over three decades. The linear extrapolation could be employed to accurately tailor the pulse width sent to the MOSFET and, therefore, the remaining QTF energy after the partial damping.

By implementing a MOSFET-based circuit, we have been able to demonstrate the active control of the damping circuit fulfilling two features: no parasitic re-excitation upon switching and controlled timing. We were able to exploit the controlled timing to realize an accurate partial damping of the QTF. The damping circuit is now suitable to be integrated for a fast characterization of the QTF in a RT-QEPAS experiment.

## 5. Fast Characterization of the QTF in a QEPAS Experiment

**Figure 8:**
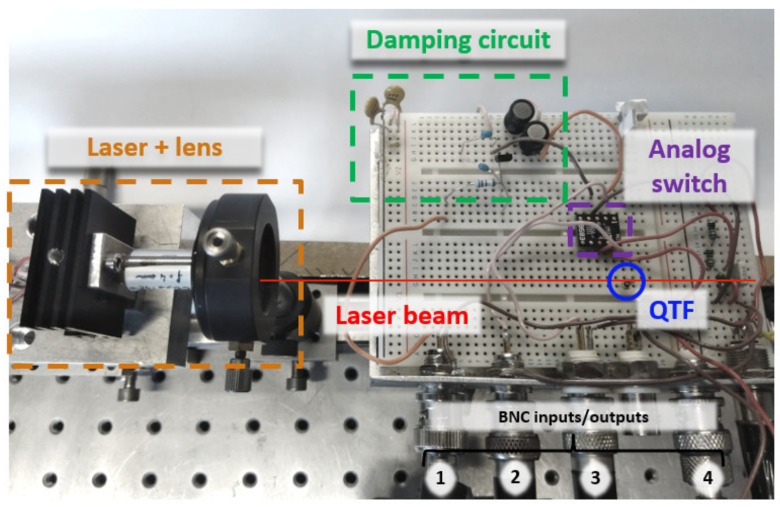
Picture of the setup-based on a standard “bare” on-beam Quartz-Enhanced PhotoAcoustic Spectroscopy (QEPAS) setup, plus the damping circuit. The QEPAS system is composed of a laser, a lens (*f* = 4 mm) and a QTF. The QTF is plugged into a test breadboard along with the other electrical components. The analog switch (controlled by BNC3) allows to switch between the QTF electrical excitation (BNC4) and the damping circuit. The damping circuit is the parallel RLC circuit resonantly matched to the QTF central frequency (see previous sections) and is disabled through applying a voltage on the MOSFET gate (BNC1). The output of the QTF (BNC2) is connected to a transimpedance amplifier and a lock-in amplifier (not represented here).

In this paragraph, we present the proof of concept of the RT-QEPAS technique implemented with the damping circuit. The setup is shown in Figure 8. The optical system is made of a laser (EBLANA Photonics 1392, Dublin, Ireland), emitting around 1.39 μm. which matches with a water vapor absorption line (7181.14 cm^−1^), and a short-focal lens (*f* = 4 mm), in order to focus the laser beam between the QTF prongs, i.e., in a on-beam configuration. No microresonators were employed for this proof of concept. The laser current is 1f wavelength-modulated at the QTF resonant frequency for the photoacoustic generation. The QTF is connected to the electronic circuit, including an analog switch (Analog Devices ADG736, Wilmington, MA, USA) for switching between the electronic excitation and the damping circuit. The analog switches have the same role than the relay (Figure 3) but exhibit lower switching parasitic currents. The damping circuit is the parallel RLC filter, controlled by a MOSFET, as schematized in Figure 6a. An important consideration for the experiment is the parasitic electromagnetic radiation due to the electrical source oscillating at a frequency close to QTF resonant frequency. The electrical excitation was turned off directly from the function generator (and not only with the analog switch), therefore preventing radiation at the breadboard level. It was experimentally verified that the QTF reached thermal noise during relaxation.

**Figure 9:**
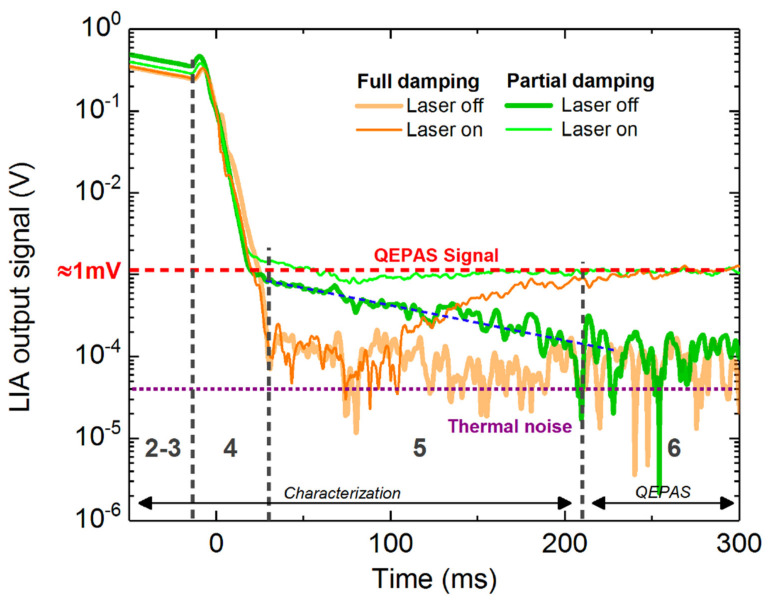
Monitoring the lock-in amplifier signal during the RT-QEPAS cycle, which is made of 6 steps: (1) excite the QTF electrically, (2) record the spontaneous relaxation to (3) find *f*_0_ and *Q*, (4) force the QTF to discharge, (5) adjust the laser modulation frequency and let the QEPAS signal to stabilize, (6) measure the gas concentration (only if the laser is on). The measurement is realized in the case of total damping (orange) and partial damping (green). For each case, the measurement is shown with the laser turned off (thick line) and turned on (thin line). For the partial damping, the MOSFET pulse was set to 40 ms. The QTF-free relaxation exponential decay is indicated by the blue dashed line (visible only on the partial damping with the laser off). The expected QEPAS signal (in this experiment) and the thermal noise are represented by the red dashed line (equivalent to 1 mV) and the purple dotted line, respectively.

A typical RT-QEPAS cycle can be observed in Figure 9. It consists of 6 steps: the steps 1 to 5 correspond to the QTF characterization, and the step 6 is the QEPAS measurement. The cycle is presented for four cases: with full damping (orange) or partial damping (green), and with laser on (thin line) or laser off (thick line). After the electrical excitation (step (1), not represented in the figure), the QTF undergoes a spontaneous relaxation (step (2)) with a typical slope of 10 ms/dec and an oscillation frequency of $f_{0}-f_{demod}$ (obtained with the in-phase signal of the LIA [15], not represented here), which are measured to calculate the QTF parameters (step (3)). The details of the method have been laid in [7]. Right after, the damping circuit is enabled to quickly discharge the QTF (step (4)). It takes about 50 ms to reach the thermal noise (purple dotted line). After the damping, the laser modulation frequency is adjusted to the value of *f*_0_ (measured at step (3)). With the laser off, the signal stays at the noise floor, while with the laser on, the QTF signal increases due to photoacoustic generation. About 200 ms are required for the QTF to charge (step (5)). Ultimately, the QEPAS signal (red dashed line) can be measured in order to deduce the gas concentration. The QEPAS signal can also be normalized by the quality factor, since the QEPAS signal is known to be proportional to *Q* [17].

It is clearly visible that the limiting stage in the cycle is the QTF charge time step, which takes about 200 ms (5). Since the damping circuit is a passive circuit, it cannot be used to provide a fast charge the same way as it is employed for a fast discharge. However, we demonstrated in Section 4 the possibility to make a partial damping. In partial damping, the expected value of the QEPAS signal can be guessed from the previous measurement and used to calculate the damping time thanks to the linear law that we extrapolated earlier (Figure 7b). The partial damping allows to partially discharge the QTF at an energy level close to the one under photoacoustic excitation, as it is observed experimentally in Figure 9 (green). When the laser is off, it can be seen that the QTF undergoes an exponential decay (blue dotted line, linear curve on log scaled graph) with a similar slope to step 2, i.e., indicating the QTF is partially charged and follows a spontaneous decay. When the laser is on, the partially charged QTF reaches the QEPAS signal very quickly.

During this experiment, the partial damping seems to perform better than the total damping. However, after partial damping the QTF oscillation phase may differ from the photoacoustic excitation phase, resulting in a destructive interference, that could lead to longer settling time. However, it was not observed experimentally. Further experimentation and simulations would be necessary to fully understand the partial damping and obtain both the fastest damping time and the fastest settling time. This proof of concept does not allow us to quantify the improvement of the charge time using partial damping compared to full damping. But the conclusion is straightforward: the partial damping has the potential to improve the charge time, most particularly when the QEPAS signal is well above the thermal noise.

## 6. Conclusions

RT-QEPAS was developed in order to tackle an important issue in QEPAS—the sensor signal drift due the variations of the QTF resonance. The method is simple and cost-effective. However, the main limitation is the long relaxation time during the QTF characterization. In this work, we proposed an improvement of RT-QEPAS based on a passive RLC damping circuit, which is resonantly matched with the QTF to quickly absorb its energy during relaxation. First, we theoretically studied the damping circuit and optimized the damping resistor and inductor. Then, we validated the optimization both by means of simulation and experiment, obtaining a relaxation time approximately 70 times faster than the lone QTF. We also developed a MOSFET-based circuit to control the activation of the damping circuit without parasitic re-excitation of the QTF. Finally, we conducted the proof of concept of the RT-QEPAS technique implemented with the damping circuit.

The damping circuit has been thoroughly optimized but its implementation in RT-QEPAS can be further improved, notably by having a better understanding of the QTF behavior during the partial damping.

In summary, the damping circuit is only made with six cheap and basic components, therefore not increasing the overall cost of the sensor but seriously improving the effectiveness of the RT QEPAS technique.
